# HMGA2 interacts with KAT6A to regulate MMPs chromatin architecture and promote triple-negative breast cancer metastasis

**DOI:** 10.3389/fimmu.2025.1590368

**Published:** 2025-05-22

**Authors:** Lu Qiao, Zenghua Liang, Pengyi Ma, Shanshan Zhang, Cuiyun Sun, Wenjun Luo, Lin Yu

**Affiliations:** ^1^ Department of Biochemistry and Molecular Biology, School of Basic Medical Sciences, Tianjin Medical University, Tianjin, China; ^2^ Laboratory of Molecular Immunology, Research Center of Basic Medical Science, Tianjin Medical University, Tianjin, China; ^3^ Tianjin Key Laboratory of Cellular and Molecular Immunology and Key Laboratory of the Educational Ministry of China, Tianjin Medical University, Tianjin, China; ^4^ Department of Radiology, Tianjin Medical University General Hospital, Tianjin, China; ^5^ Department of Neuropathology, Tianjin Key Laboratory of Injuries, Variations and Regeneration of the Nervous System, Key Laboratory of Post-trauma Neuro-repair and Regeneration in Central Nervous System of Education Ministry, Tianjin Neurological Institute, Tianjin Medical University General Hospital, Tianjin, China

**Keywords:** triple-negative breast cancer, high mobility group AT-hook 2, matrix metalloproteinases, lysine acetyltransferase 6A, metastasis

## Abstract

**Background:**

Triple-negative breast cancer (TNBC), the most lethal breast cancer subtype, demonstrates poor prognosis due to its high rates of metastasis, recurrence, and mortality. The metastatic potential in TNBC patients serves as a critical determinant of clinical outcomes. The high mobility group AT-hook 2 (HMGA2) has emerged as a novel chromatin architectural regulator, its specific role in TNBC metastasis requires further investigation.

**Methods:**

We analyzed the expression of HMGA2 in TNBC and non-TNBC patients using Genomic Data Commons (GDC) The Cancer Genome Atlas (TCGA) and immunohistochemistry. The correlation between HMGA2 expression and patient prognosis was assessed using the Kaplan-Meier estimator. The roles of HMGA2 in TNBC metastasis were validated through cell wound healing assay, transwell assay and lung metastatic model. RNA sequencing, chromatin immunoprecipitation, DNA electrophoretic mobility shift, co-immunoprecipitation and chromosome conformation capture assays were applied to identify the mechanisms by how HMGA2 functions as a novel chromatin architectural regulator.

**Results:**

Our study revealed significantly upregulated HMGA2 expression in TNBC patients compared to non-TNBC patients. Kaplan-Meier survival analysis demonstrated a strong association between elevated HMGA2 expression and poor prognosis in TNBC cases. Functional studies showed that HMGA2 downregulation markedly inhibited TNBC metastatic progression. Mechanistic investigations revealed that HMGA2 facilitates TNBC metastasis through transcriptional activation of matrix metalloproteinases (MMPs). Specifically, HMGA2 interacted with lysine acetyltransferase 6A (KAT6A) to mediate histone acetylation at *MMPs* promoter regions. Concurrently, HMGA2 induced chromatin conformation changes to enhance MMPs transcriptional activity.

**Conclusion:**

These findings establish that the HMGA2/KAT6A complex promote MMPs expression to drive TNBC metastasis, identifying novel therapeutic targets for this aggressive malignancy.

## 1 Introduction

Breast cancer is the most frequently diagnosed malignancy among women worldwide, with metastasis being the leading cause of mortality (1). Among its subtypes, triple-negative breast cancer (TNBC) is the most aggressive; it exhibits high metastatic potential, elevated recurrence rates, and poor prognosis (2). A comprehensive understanding of the molecular mechanisms underlying TNBC metastasis is crucial for advancing targeted interventions.

High mobility group AT-hook 2 (HMGA2) functions as a non-histone architectural transcription factor (3). Its DNA-binding domain is located at the N-terminal of HMGA2, whereas the acidic domain resides at the C-terminal (4). HMGA2 specifically binds to AT-rich DNA sequences, thereby modifying the chromatin architecture and regulating the transcription of target genes. It participates in diverse biological processes through protein-protein interactions (5). During embryogenesis, HMGA2 expression is significantly elevated but remains relatively low in most adult tissues. However, its overexpression is frequently observed in various human malignancies, thus indicating its potential oncogenic role (6). For example, HMGA2 binds to the signal transducer and activator of transcription 3 (*STAT3*) promoter to activate its transcription and promote macrophage recruitment in colorectal cancer (CRC) (7). HMGA2 also induces transforming growth factor beta 2 (TGFβ-2) receptor expression, activates the TGFβ signaling pathway, and facilitates cancer cell metastasis (8). Accumulating evidence identifies HMGA2 as a key driver of epithelial-to-mesenchymal transition (EMT) (9). Additionally, its interaction with retinoblastoma protein (pRb) mediates forkhead box L2 (FOXL2) transactivation and potentially contributes to metastatic progression (10). Moreover, HMGA2 interacts with poly(ADP-ribose) polymerase 1 (PARP1) by enhancing PARP1 activity and reducing cancer cell sensitivity to olaparib (a PARP inhibitor) (11). Previous studies have demonstrated HMGA2’s role in TNBC proliferation, migration, and invasion (12, 13). However, its precise function as a chromatin architectural regulator in TNBC metastasis remains unclear.

Lysine acetyltransferase 6A (KAT6A), a MYST family member, regulates gene expression by acetylating target gene promoters and enhancers (14). KAT6A specifically mediates histone acetylation at H3 lysine 9 (H3K9) (H3K9ac) and H3K23 (H3K23ac) (15). Substantial evidence supports its oncogenic role in various cancers. For instance, KAT6A regulates stemness and proliferation (16). In ovarian cancer, it binds to and acetylates constitutive photomorphogenesis 1 (COP1) at K294, resulting in β-catenin accumulation and enhanced activity (17). In glioma, KAT6A activates phosphatidylinositol-4,5-bisphosphate 3-kinase catalytic subunit alpha (PIK3CA) transcription by recruiting tripartite motif-containing 24 (TRIM24), thereby promoting tumorigenesis (18).

Matrix metalloproteinases (MMPs), members of the metzincin protease superfamily (19), are frequently upregulated in cancers (20). These proteolytic enzymes drive cancer progression by degrading the extracellular matrix, facilitating angiogenesis, and shaping the tumor microenvironment (21). Previous studies have demonstrated that MMP1 enhances cancer invasion and metastasis (22) while also regulating cancer stemness and chemoresistance (23). MMP2, activated by MMP1 (24), with MMP9, specifically degrade the extracellular matrix, thereby facilitating metastasis (25).

This study demonstrates that HMGA2 is significantly overexpressed in TNBC and correlates with poor prognosis. Mechanistically, the study identifies KAT6A as a novel HMGA2-interacting partner, which forms a complex that binds to *MMPs* promoters and catalyzes site-specific histone acetylation. Furthermore, HMGA2 induces chromatin conformation changes at *MMPs* promoter regions and directly enhances MMPs transcription. The resulting MMPs upregulation promotes extracellular matrix degradation and drives TNBC metastasis.

## 2 Materials and methods

### 2.1 Database analysis

Gene expression data for breast cancer were obtained from Genomic Data Commons (GDC) The Cancer Genome Atlas (TCGA) (https://gdc.xenahubs.net). The 20 datasets included 1,104 tumor and 113 normal tissues, with tumor subtypes categorized as follows: 616 Luminal, 67 human epidermal growth factor receptor 2 (HER2)-positive, and 142 TNBC. Pearson’s correlation analysis between HMGA2 and MMPs messenger ribonucleic acid (mRNA) expression levels was performed using the online platform www.bioinformatics.cn.

### 2.2 Clinical information and tissue specimens

Human tissue specimens, including 6 normal, 60 TNBC, and 20 non-TNBC cases, were collected from the Tianjin Medical University General Hospital (TMUGH). The study was approved by the Ethics Committee of TMUGH. The ethics lot number is IRB2020-KY-289. Detailed patient information is provided in **Supplementary Table 1**.

### 2.3 Cell culture

MDA-MB-231 and BT549 cells were obtained from the China Center for Type Culture Collection (CCTCC) and authenticated by short tandem repeat (STR) profiling. HEK293T cells were purchased from the American Type Culture Collection (ATCC). MDA-MB-231 and HEK293T cells were cultured in Dulbecco’s modified Eagle’s medium (DMEM) supplemented with 10% fetal bovine serum (FBS). BT549 cells were maintained in Roswell Park Memorial Institute 1640 (RPMI-1640) medium supplemented with 10% FBS and 1 μg/mL insulin. All cells were cultured at 37°C with 5% CO_2_ in a humidified incubator.

### 2.4 Lentivirus packaging

Two HMGA2 short hairpin (sh) RNA plasmids were constructed as TRC2-pLKO-shHMGA2-1 (5’-AGTCCCTCTAAAGCAGCTCAA-3’) and TRC2-pLKO-shHMGA2-2 (5’-AGGAGGAAACTGAAGAGACAT-3’). The full-length human HMGA2 sequence, HMGA2 deletion mutant (D1, D2, D3) sequence were cloned into the pLVX-IRES-puro expression vector to generate pLVX-IRES-HMGA2, pLVX-IRES-HMGA2-D1, pLVX-IRES-HMGA2-D2, pLVX-IRES-HMGA2-D3. All plasmids were constructed by GENEWIZ. When the fusion degree of HEK293T cells reached about 70%, transient transfection of expression plasmids along with packaging plasmids using polyethyleneimine (PEI). The viral supernatant was collected at 24 h and 48 h, centrifuged for removal of floating cells, filtered through a 0.45 µm filter. MDA-MB-231 and BT549 cells were infected with the lentiviruses in the presence of 1 µg/mL puromycin. The screened cells were diluted in cascaded gradients and cultured in 96-well plates. After about 7–14 days, monoclonal formation could be see under the microscope. Selected a cell population with only one monoclonal and expanded the culture to obtain a stable knockdown or overexpressed cell lines.

### 2.5 Immunohistochemistry (IHC)

IHC was performed as previously described (26). A polyclonal HMGA2 antibody was applied to the tissues at a 1:250 dilution.

### 2.6 Western blotting (WB)

The lysis solution contained radioimmunoprecipitation assay (RIPA) lysis buffer (Solarbio, R0010), 1 mM phenylmethylsulfonyl fluoride (PMSF) (Solarbio, P0100), and 1 × protease inhibitor cocktail (PIC) (Solarbio, P6730). Protein concentrations were measured using a bicinchoninic acid assay (BCA) protein assay kit (Thermo Scientific, A55864). Proteins were separated by sodium dodecyl sulfate-polyacrylamide gel electrophoresis (SDS-PAGE) and transferred onto a nitrocellulose (NC) membrane (PALL, 66485). After blocking with 5% skim milk (Solarbio, D8340) for approximately 2 h, NC membranes were incubated with primary antibodies at 4 °C for 12–16 h. The next day, NC membranes were washed with tris-buffered saline with tween 20 (TBST) to remove residual primary antibodies, followed by incubation with secondary antibodies for 1–2 h. Protein bands were visualized using a chemiluminescence detection system. The primary antibodies used in WB were anti-HMGA2 (Proteintech, 20795-1-AP), anti-MMP1 (Proteintech, 10371-2-AP), anti-MMP2 (Proteintech, 10373-2-AP), anti-MMP9 (Proteintech, 10375-2-AP), anti-KAT6A (Santa Cruz Biotechnology, sc-293283), anti-Tubulin (Proteintech, 11224-1-AP), anti-Flag (Proteintech, 20543-1-AP).

### 2.7 Real-time quantitative polymerase chain reaction

Total RNA was extracted using RNA Isolator Total RNA Extraction Reagent (Vazyme, R401-01), and 1 μg of cDNA was synthesized with a reverse transcription kit (GenStar, A236-04). Quantitative polymerase chain reaction (qPCR) was performed using 2 × RealStar Fast SYBR qPCR Mix (GenStar, A303). The primers used are listed in **Supplementary Table 2**.

### 2.8 Immunofluorescence (IF)

TNBC cells were fixed with 4% paraformaldehyde (Solarbio, P1110) for 10 min, permeabilized with 0.5% Triton X-100 (Sigma-Aldrich, 93443) about 5 min and blocked with 5% bovine serum albumin (BSA) for 1 h at room temperature. Cells were incubated in primary antibody solution at 4°C overnight. HMGA2 was detected using a rabbit polyclonal antibody (dilution ratio 1: 50). Cells were rewarmed at room temperature for 1 h. After washing five times with phosphate buffered saline (PBS) (Solarbio, P1010), cells were incubated with goat anti-rabbit IgG (H + L) highly cross-adsorbed secondary antibody, Alexa Fluor™ 488 (Invitrogen, A-11034, dilution ratio 1: 200). Finally, cells washed five times with PBS and nuclei were stained with DAPI (Sigma-Aldrich, F6057).

### 2.9 Cell wound healing assay

TNBC cells were seeded in six-well plates and allowed to adhere. A perpendicular scratch was made, and the medium was replaced with 1% FBS. Images were captured at 0 h and 24 h using a microscope, whereas migration was quantified with ImageJ.

### 2.10 Transwell assay

Matrigel matrix (Corning, 356234) was diluted to an appropriate concentration and pre-added to the upper chamber of Transwell inserts (Millipore, PTEP24H48), followed by incubation at 37°C for 1 h. Approximately 1 × 10^5^ TNBC cells were resuspended in 100 μL medium (with 0% FBS) and then seeded into the upper chamber. The lower chamber was filled with appropriate medium (10% FBS). After 12–16 h of incubation, cells that invaded the inferior surface of the membrane were fixed with 4% paraformaldehyde and stained with crystal violet using ImageJ software to calculate relative invasion.

### 2.11 Lung metastatic model

Animal experiments strictly followed the NIH Principles for the Care of Laboratory Animals and were approved by the Institutional Animal Care and Use Committee of Tianjin Medical University. The review number is TMUaMEC 2023012. Six-week-old female BALB/c nude mice were used to establish a lung metastatic model. Approximately 1 × 10^6^ MDA-MB-231 cells resuspended in 150 μL of PBS were injected into mice tail vein (n = 5). Metastasis was monitored by bioluminescence imaging after intraperitoneal injection of D-luciferin potassium salt (Beyotime, ST196) using a *In Vivo* Imaging System (IVIS).

### 2.12 RNA sequencing

The experiment was performed using shNC or shHMGA2 from MDA-MB-231 cells and sequenced using GENEWIZ. Differentially expressed genes (DEGs) were analyzed using DESeq2 (V1.26.0) in the Bioconductor software package.

### 2.13 Gelatin zymography assay

When the fusion rate of TNBC cells reached 70 – 80%, the original medium was removed and replaced with a serum-free medium for further culture. The conditioned medium was collected at 12 h and 24 h, concentrated, and dissolved in 5 × non-reducing buffer. Proteins were separated on a 7.5% acrylamide gel containing gelatin, and the gel was washed and stained to visualize the gelatinolytic activity as white bands against a blue background.

### 2.14 Chromatin immunoprecipitation (ChIP)

A sonication ChIP kit (ABclonal, RK20258) was used to perform ChIP assays. Briefly, cells were crosslinked using a 1% formaldehyde solution (Sigma, F8775), and glycine was added to terminate crosslinking. Chromatin was sheared by sonication and 5% of the lysate was saved as an input. Then the target protein antibodies were added to the remaining ultrasound-treated sample for 12–16 h at 4 °C. The normal rabbit IgG antibody (Cell Signaling Technology, #2729) or mouse IgG antibody (Proteintech, B900620) were added to the negative control group. Protein-DNA complexes were captured using ChIP-grade magnetic beads (ABclonal, RM02915), eluted, and crosslinked. Target DNA enrichment was quantified using qPCR. The primary antibodies used in ChIP were anti-HMGA2, anti-H3K23ac (abcam, ab177275). The ChIP primers are listed in **Supplementary Table 3**.

### 2.15 Electrophoretic mobility shift assay (EMSA)

Biotin-labeled and unlabeled probes corresponding to the *MMPs* promoter sequences were synthesized using GENEWIZ. A Chemiluminescent EMSA Kit (Beyotime, GS009) was used to complete the EMSA assay according to the manufacturer’s protocol. The reaction groups included: 1) negative control (biotin-labeled probe only), 2) binding reaction (biotin-labeled probe + nuclear protein), and 3) competition reaction (biotin-labeled probe + nuclear protein + increasing concentrations of unlabeled probe). Samples were electrophoresed on an 8% polyacrylamide gel, transferred to a positively charged nylon membrane (Beyotime, FFN10), and crosslinked under UV light (120 mJ/cm² for 2 min). Probes were detected using horseradish peroxidase-coupled streptavidin. The EMSA probe sequences are listed in **Supplementary Table 4**.

### 2.16 Immunoprecipitation and co-immunoprecipitation (Co-IP)

Cells were lysed in NP-40 Lysis Buffer (Beyotime, P0013F) with PMSF and PIC for 1 h at 4 °C. The lysates were centrifuged at 12,000 rpm for 15 min. Overall, 100 μL of the supernatant was saved as the input. The remaining supernatant was incubated with IgG (negative control) or target protein antibodies for 12–16 h at 4°C. Added protein A/G magnetic beads (Selleck, B23201) approximately 3 h at 4 °C. IP wash buffer was used to wash the magnetic beads approximately six times and then 2 × loading buffer was used to resuspend the magnetic beads. For IP, the samples were analyzed using Coomassie Brilliant Blue staining. For Co-IP, the samples were analyzed by WB. The primary antibodies used in Co-IP were anti-HMGA2, anti-KAT6A, anti-Flag.

### 2.17 Chromosome conformation capture (3C) assay

Approximately 1 × 10^7^ cells were crosslinked with 1% formaldehyde to fix the chromatin conformation, and glycine was added to fully terminate formaldehyde crosslinking. Nuclei were lysed in 3C lysis buffer and digested with DpnII (NEB, R0543S) at 37°C for 12–16 h. The digested fragments were ligated using T4 DNA ligase (NEB, M0202S) at 16°C for 8 h. The ligation efficiency between the anchor sites and other sites was quantified by qPCR. Standard samples were generated by amplifying *MMPs* promoter regions, digesting with DpnII, and ligating with T4 DNA ligase. The crosslinking rates were normalized to the standard. All the primers used are listed in **Supplementary Table 5**.

### 2.18 Statistical analysis

GraphPad Prism 8.0.1 software was used for statistical analysis of experimental data. Student’s t-test or one-way ANOVA were used to compare the statistical significance. All the results were presented as the mean ± standard deviation (SD) from three independent experiments. *p<0.05, **p<0.01, ***p<0.001, ****p<0.0001 was regarded as indicative of statistical significance.

## 3 Results

### 3.1 HMGA2 is highly expressed in TNBC and associates with patients’ poor prognosis

**Figure 1:**
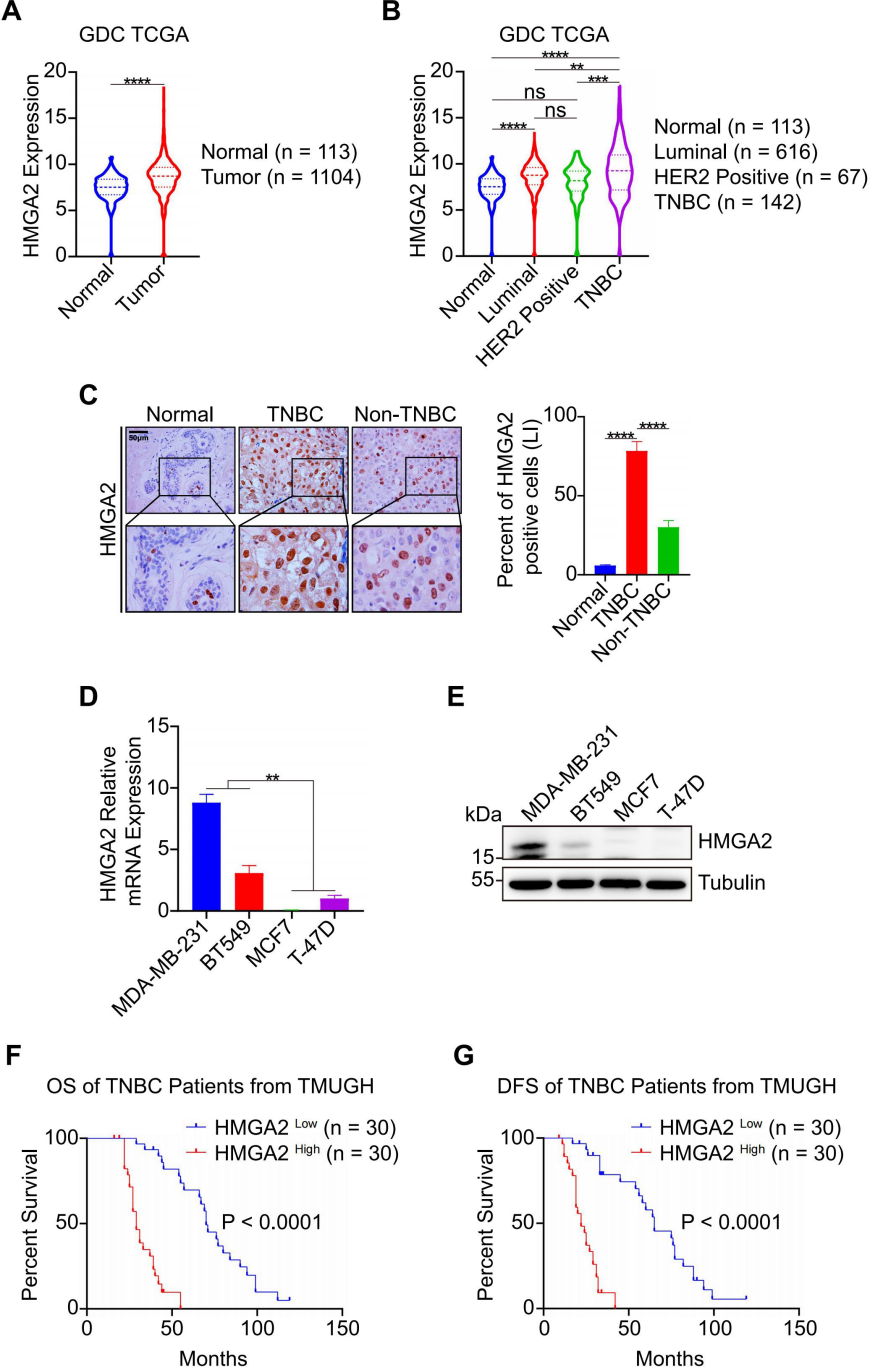
HMGA2 is highly expressed in TNBC and associates with patients’ poor prognosis. **(A)** HMGA2 mRNA expression levels in normal and tumor tissues. Data were obtained from GDC TCGA. **(B)** HMGA2 mRNA expression levels in TNBC and non-TNBC tissues. Data were obtained from GDC TCGA. Breast cancer can be classified according to their expression of estrogen receptor (ER), progesterone receptor (PR), HER2 and Ki67. **(C)** The representative HMGA2 IHC images of the FFPE samples, 6 cases of normal tissues, 60 cases of TNBC tissues and 20 cases of non-TNBC tissues. Scale bar: 50 μm. Magnification: 400 ×. **(D, E)** mRNA **(D)** and protein **(E)** of HMGA2 expression in TNBC cells (MDA-MB-231 and BT549) were higher than those of MCF7 and T-47D cell lines. **(F, G)** TNBC patients with high HMGA2 expression exhibited poor prognosis. TNBC patients from TMUGH. ns, no significance; **p<0.01, ***p<0.001, ****p<0.0001.

To investigate HMGA2 expression patterns in breast cancer tissues, we analyzed GDC TCGA (https://gdc.xenahubs.net), which revealed significantly elevated HMGA2 mRNA levels in tumor tissues than in normal tissues (**Figure 1A**). Subtype-specific analysis showed that TNBC samples exhibited the highest HMGA2 expression levels (**Figure 1B** which was further validated by IHC staining (**Figure 1C**). Comparative analysis across breast cancer cell lines revealed significantly higher HMGA2 expression in TNBC cell lines than in MCF7 and T-47D cell lines (**Figures 1D, E**). At the same time, the endogenous HMGA2 in TNBC cells were detected by IF (**Supplementary Figure 1A**).

To assess HMGA2’s prognostic value, we performed Cox proportional hazard modeling in patients with TNBC. Both univariate and multivariate analyses identified TNM stage, MKI67 expression, and HMGA2 levels as prognostic factors for overall survival (OS) and disease-free survival (DFS) in the TMUGH cohort (**Tables 1**, **2**). Survival analysis of patients with TNBC from TMUGH cohort (**Figures 1F, G**) showed that elevated HMGA2 expression correlated with significantly shorter OS and DFS. Collectively, these findings establish high HMGA2 expression as a potential indicator of poor prognosis in patients with TNBC.

**Table 1:** Univariate analysis for DFS and OS in our TNBC samples.

Factors	DFS		OS	
	HR (95% CI)	*P*	HR (95% CI)	*P*
Age	1.214 (0.916-1.375)	0.087	1.259 (1.113-1.325)	0.029
TNM stage	1.335 (1.292-1.401)	0.008	1.252 (1.173-1.452)	<0.001
MKI67 LI	1.192 (1.088-1.214)	0.007	1.219 (1.188-1.304)	0.019
HMGA2 LI	1.175 (1.110-1.251)	<0.001	1.207 (1.189-1.254)	<0.001

HR, hazard ratio; CI, confidence interval; LI, labeling index.

**Table 2:** Multivariate analysis for DFS and OS in our TNBC samples.

Factors	DFS		OS	
	HR (95% CI)	*P*	HR (95% CI)	*P*
Age	1.129 (0.892-1.277)	0.072	1.201 (1.092-1.357)	0.044
TNM stage	1.392 (1.217-1.407)	<0.001	1.194 (1.131-1.510)	<0.001
MKI67 LI	1.141 (1.077-1.235)	0.022	1.232 (1.192-1.276)	0.007
HMGA2 LI	1.194 (1.163-1.271)	<0.001	1.244 (1.192-1.281)	<0.001

HR, hazard ratio; CI, confidence interval; LI, labeling index.

### 3.2 HMGA2 promotes the metastasis of TNBC

**Figure 2:**
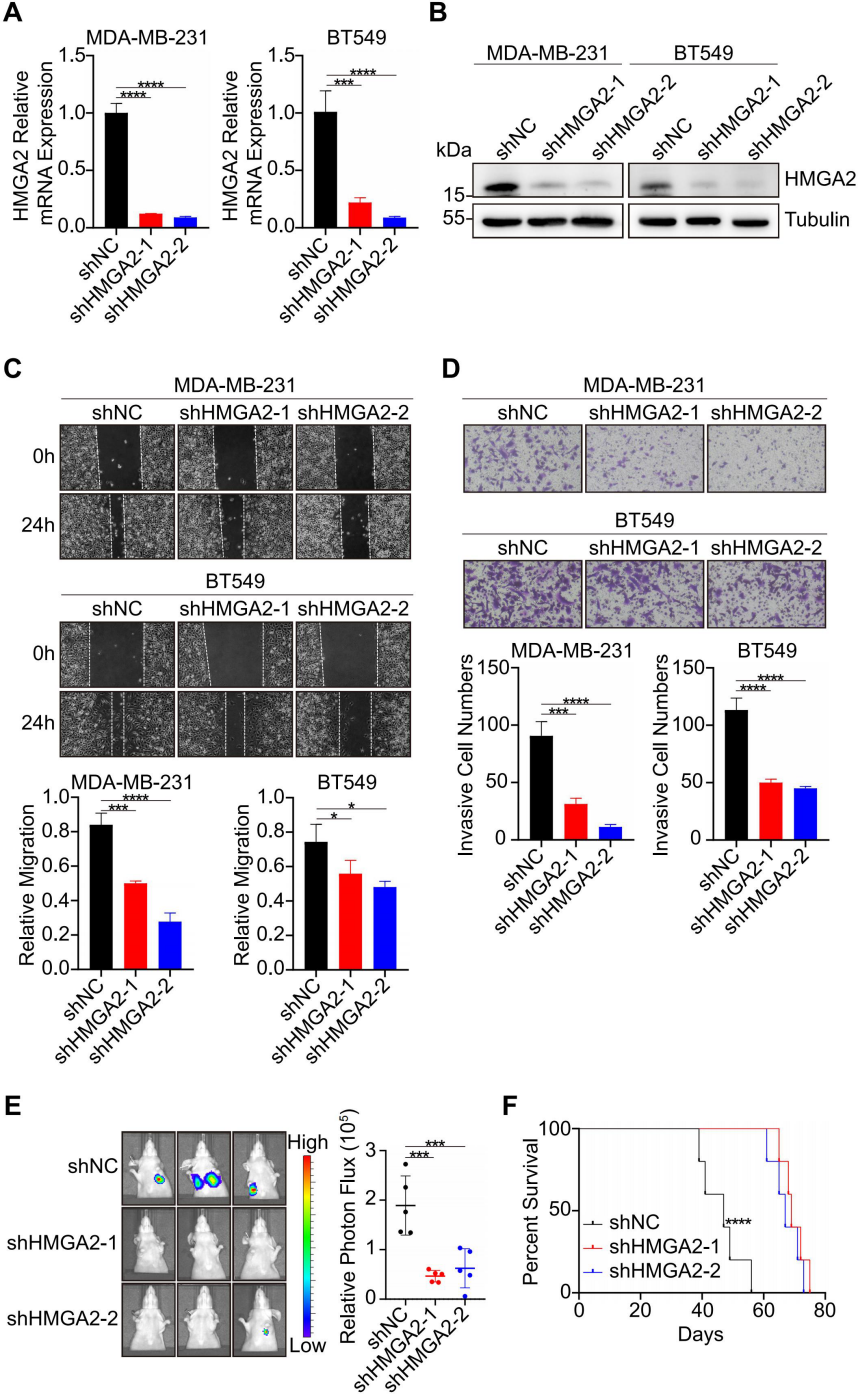
HMGA2 promotes the metastasis of TNBC. **(A, B)** RT-qPCR **(A)** and WB **(B)** were performed to assess the knockdown efficiency of HMGA2. **(C)** Wound healing assays demonstrated HMGA2 knockdown inhibited the migration in TNBC cells. **(D)** Transwell assays demonstrated HMGA2 knockdown inhibited the invasion in TNBC cells. **(E)** Representative images of lung metastasis in mice injected with shNC and shHMGA2 cells (n = 5). **(F)** Survival time of mice of lung metastasis with shNC and shHMGA2 cells (n = 5). *p<0.05, ***p<0.001, ****p<0.0001.

To further investigate the functional role of HMGA2 in TNBC metastasis, we established HMGA2 stable knockdown models using shRNA that specifically targeted HMGA2 (shHMGA2–1 and shHMGA2-2). Knockdown efficiency was confirmed by RT-qPCR and WB analysis (**Figures 2A, B**). Functional assays demonstrated that HMGA2 knockdown significantly inhibited the migratory and invasive capabilities of TNBC cells (**Figures 2C, D**). To evaluate *in vivo* metastatic potential, we established a lung metastatic model. Comparative analysis revealed that the shHMGA2 groups exhibited significantly reduced metastatic capacity and prolonged survival time compared to the shNC group (**Figures 2E, F**). Collectively, these findings strongly suggest that HMGA2 is essential for promoting TNBC metastasis.

### 3.3 HMGA2 regulates the expression of genes associated with TNBC metastasis

**Figure 3:**
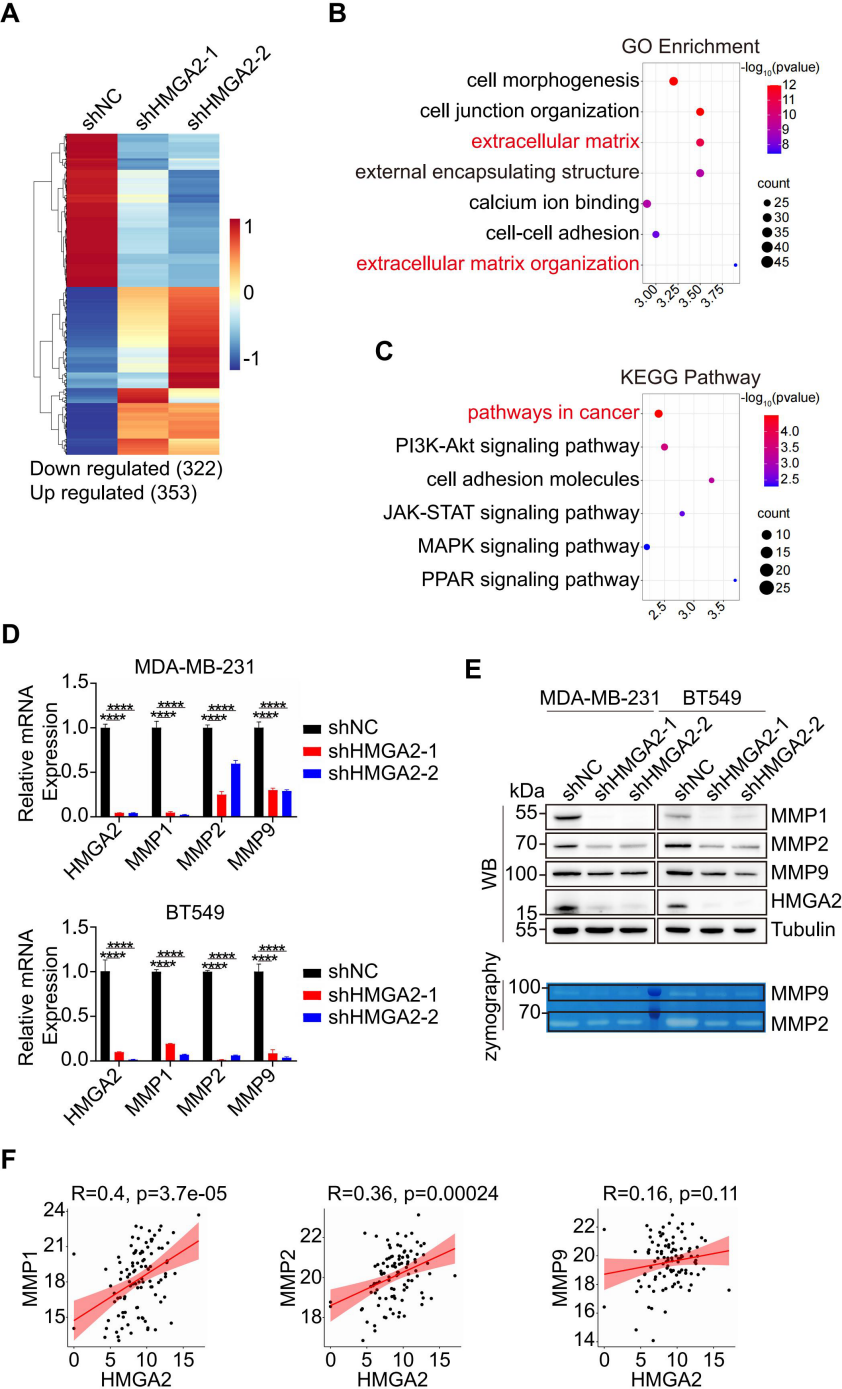
HMGA2 regulates the expression of MMPs. **(A)** Heatmap from the RNA-sequencing data showed the DEGs in HMGA2 knowdown or control cells. **(B, C)** Representative GO **(B)** and KEGG **(C)** term analysis of DEGs. **(D, E)** After HMGA2 knowdown in TNBC cells, MMPs expression levels were detected by RT-qPCR **(D)** and WB (**E**, upper part). MMP2 and MMP9 enzymatic activities were decreased following HMGA2 knockdown (**E**, lower part). **(F)** Correlation between HMGA2 and MMPs expression. Data were obtained from GDC TCGA. ****p<0.0001.

Building on our findings regarding HMGA2’s role in TNBC metastasis, we sought to elucidate the underlying molecular mechanisms. We initially performed RNA sequencing of MDA-MB-231 cells transfected with either shNC or shHMGA2 to identify DEGs. The results were screened according to the difference significance criteria, DEGs change ≥ 2-fold and false discovery rate (FDR) below 0.05. Hierarchical clustering analysis revealed 322 down regulated genes and 353 up regulated genes in shHMGA2 cells compared to shNC cells (**Figure 3A**). Subsequent Gene Ontology (GO) enrichment analysis and Kyoto Encyclopedia of Genes and Genomes (KEGG) pathway analysis of DEGs indicated that HMGA2 primarily regulates “extracellular matrix”, “extracellular matrix organization” and “pathways in cancer”, suggesting its crucial involvement in TNBC metastasis (**Figures 3B, C**).

MMPs are the key enzymes for degrading extracellular matrix, to further validate the MMPs expression patterns, we performed RT-qPCR and WB analyses. Transcriptional analysis revealed a significant downregulation of MMP1, MMP2, and MMP9 in shHMGA2 groups (**Figure 3D**). WB analysis confirmed a corresponding reduction in protein expression levels (**Figure 3E**, upper panel). Gelatin zymography assays demonstrated markedly decreased enzymatic activity of both MMP2 and MMP9 following HMGA2 knockdown (**Figure 3E**, lower panel; **Supplementary Figure 2A**). Analysis of GDC TCGA revealed positive correlations between HMGA2 and MMP1/MMP2 mRNA expression levels, with a moderate correlation observed for MMP9 (**Figure 3F**). These comprehensive findings support our hypothesis that HMGA2 promotes TNBC metastasis through functional activation of MMPs.

### 3.4 HMGA2 regulates transcription of MMPs

**Figure 4:**
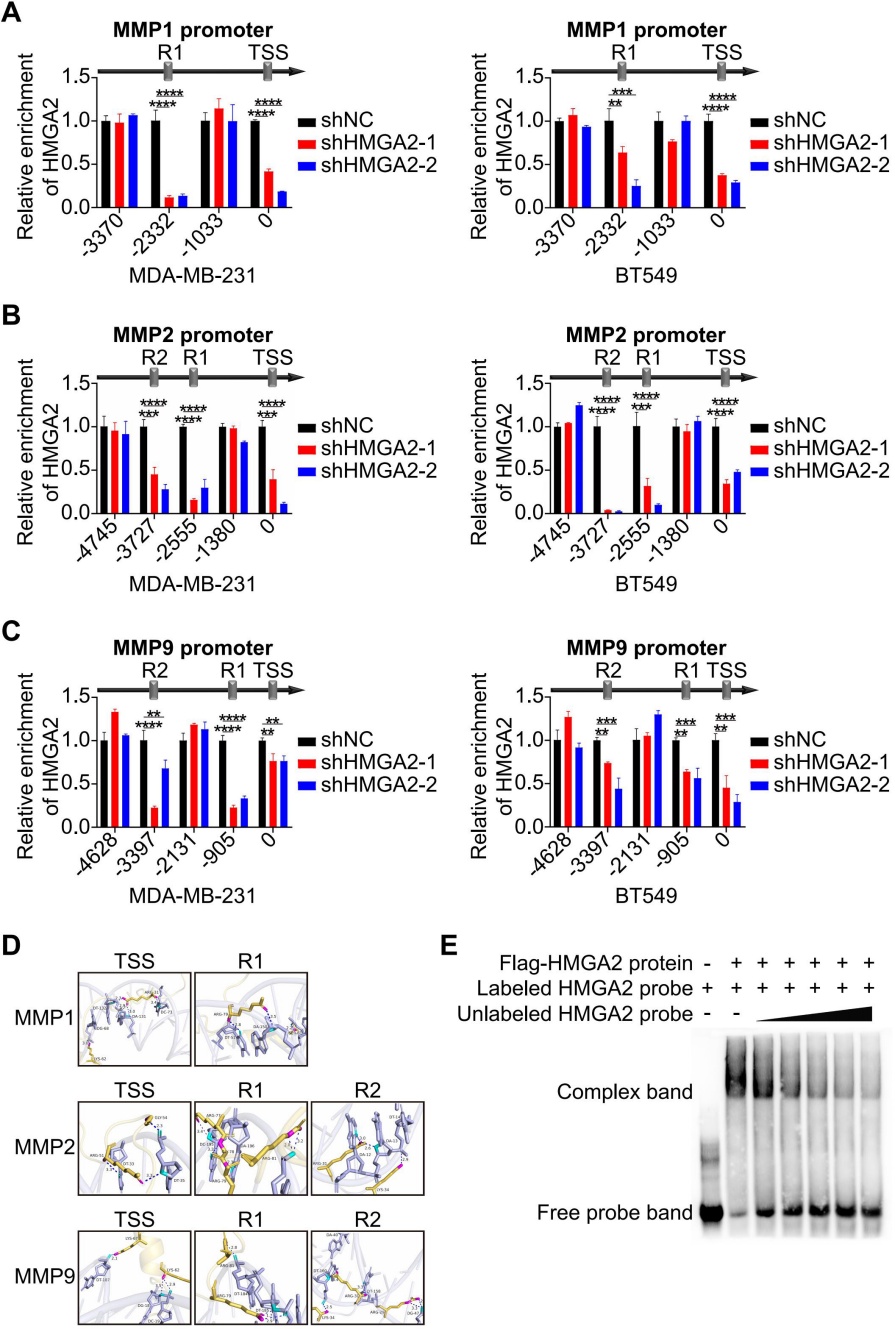
HMGA2 regulates transcription of MMPs. **(A-C)** ChIP results demonstrated the interaction of HMGA2 with promoters of *MMPs* in TNBC cells. (**D)** Schematic diagram illustrated HMGA2 binds to conserved regions at *MMPs* promoters. (**E)** Interactions between *in vitro* translated HMGA2 and *MMPs* promoters were verified by EMSA. **p<0.01, ***p<0.001, ****p<0.0001.

To identify the specific binding regions for HMGA2, we performed ChIP assays on TNBC cells. The results demonstrated HMGA2 binding at the transcription start site (TSS) and R1 (located upstream of TSS) of *MMP1*, as well as at the TSS, R1 and R2 (located upstream of R1) of both *MMP2* or *MMP9*. HMGA2 knockdown significantly reduced its interaction with these conserved regions, confirming the specificity of HMGA2 recruitment to the *MMPs* promoters (**Figures 4A–C**). Structural predictions using AlphaFold 3 supported HMGA2’s ability to bind to the TSS, R1, and R2 of these target genes (**Figure 4D**). The EMSA results showed that HMGA2 specifically bound to labeled probes (**Figures 4E**, lane 2), with unlabeled probes competitively inhibiting complex formation in a dose-dependent manner (**Figure 4E**, Lanes 3-7). Collectively, these findings demonstrate that HMGA2 directly interacts with the conserved regions of the *MMPs* promoters to regulate transcriptional activity.

### 3.5 HMGA2 interacts with KAT6A regulating the expression of MMPs

**Figure 5:**
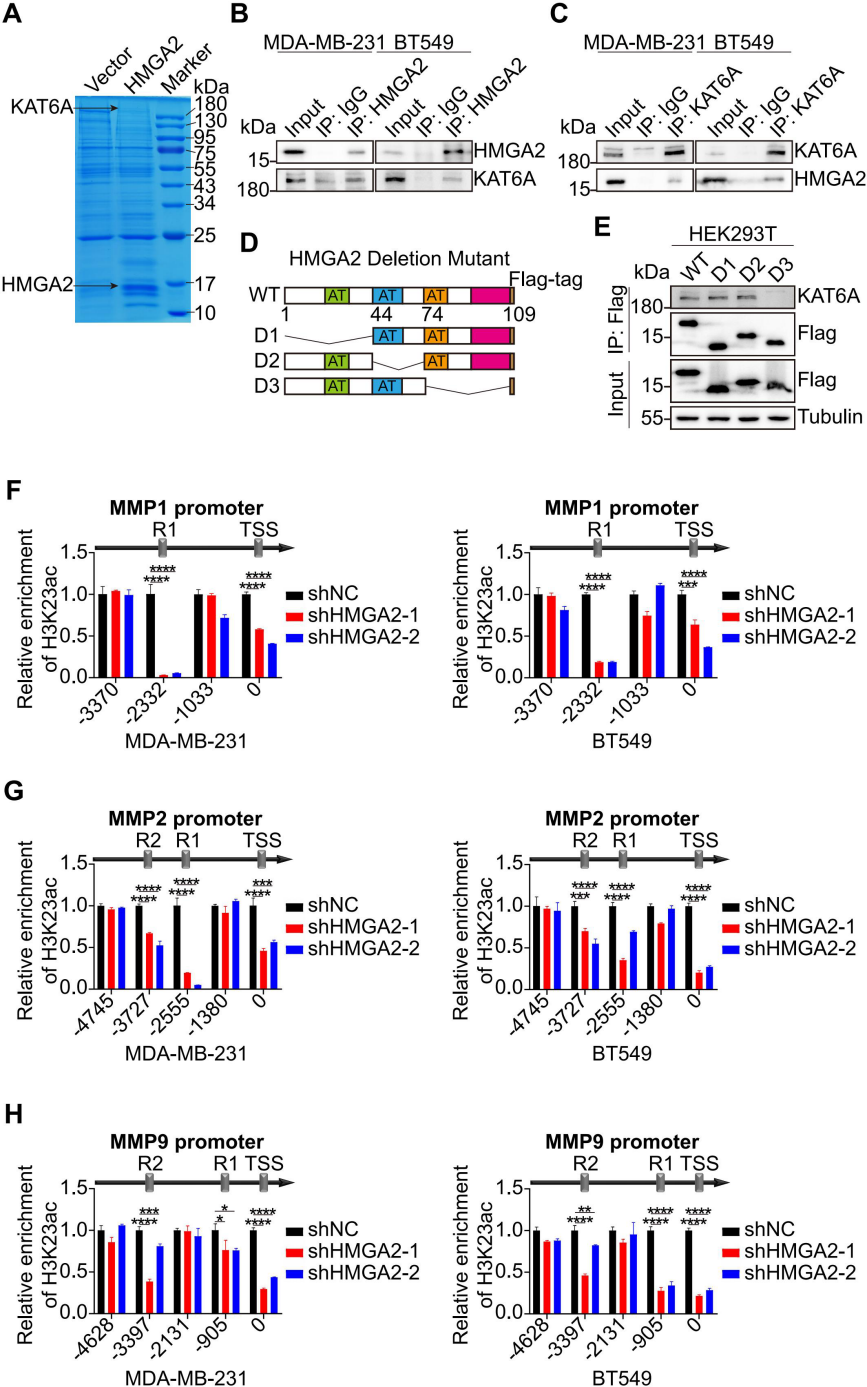
HMGA2 interacts with KAT6A regulating the expression of MMPs. **(A)** Proteins from the IP assay on MDA-MB-231 cells were separated by SDS-PAGE and detected by Coomassie brilliant blue staining. **(B, C)** Interaction between HMGA2 and KAT6A were verified in MDA-MB-231 **(B)** and BT549 **(C)** cells by Co-IP assay. **(D)** Schematic of various HMGA2 truncations. **(E)** Total cell lysates from HEK293T cells expressing different truncations of HMGA2 were subjected to IP with antibodies against Flag tags. **(F–H)** ChIP results demonstrated the interaction of H3K23ac with promoters of *MMPs* in TNBC cells. *p<0.05, **p<0.01, ***p<0.001, ****p<0.0001.

The potential interacting proteins of HMGA2 were revealed by IP experiments. Mass spectrometry (MS) analysis of the HMGA2 interactome in vector versus HMGA2 cells identified KAT6A as a potential interacting partner in TNBC cells (**Figure 5A**). Co-IP experiments confirmed the physical interaction between HMGA2 and KAT6A in TNBC cells (**Figures 5B, C**). Through the ectopic expression of various HMGA2 truncation mutants, we observed that the D3 mutant (lacking amino acids 74-109) exhibited a significantly reduced interaction with KAT6A compared to the D1 and D2 mutants (**Figures 5D, E**).

Given KAT6A’s established role in mediating H3K23ac, we performed additional ChIP assays to target this histone modification. The analysis revealed that H3K23ac binding regions coincided with HMGA2 binding regions in *MMPs* promoters, and HMGA2 silencing markedly decreased histone modification interactions at these regions (**Figures 5F-H**).

### 3.6 HMGA2 promotes MMPs transcription via chromatin conformation changes

**Figure 6:**
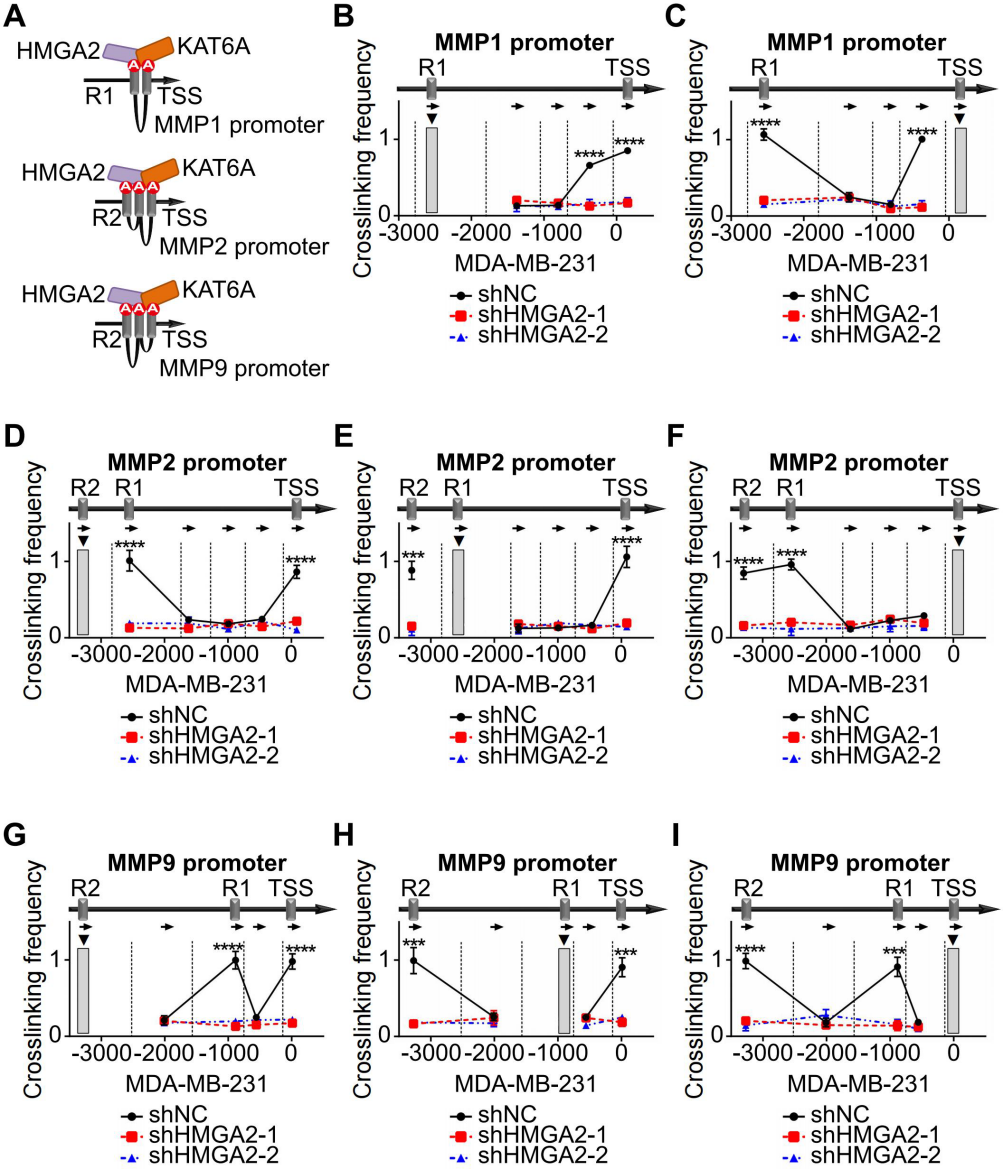
HMGA2 promotes MMPs transcription via chromatin conformation changes. **(A)** Schematic structure illustrating the mechanism of HMGA2 induced chromatin conformation changes on *MMPs* promoters. **(B)** In MDA-MB-231 cells, the anchor was R1 of *MMP1*, 3C data showed the DNA cross-link among R1 to TSS. HMGA2 knockdown attenuated the formation of R1/TSS cross-linking. Restriction enzyme cleavage sites were displayed by vertical lines, and direction of PCR primers were represented by arrows. **(C)** In MDA-MB-231 cells, the anchor was TSS of *MMP1*, and the cross-link among TSS to R1 were shown. **(D, G)** In MDA-MB-231 cells, the anchor was R2 of *MMP2* or *MMP9*, and the cross-links among R2 to R1 and TSS were shown. **(E, H)** In MDA-MB-231 cells, the anchor was R1 of *MMP2* or *MMP9*, and the cross-links among R1 to R2 and TSS were shown. **(F, I)** In MDA-MB-231 cells, the anchor was TSS of *MMP2* or *MMP9*, and the cross-links among TSS to R1 and R2 were shown. ***p<0.001, ****p<0.0001.

Based on the ChIP results, the spatial conformations of the *MMPs* promoter regions were inferred the **Figure 6A**. To investigate the role of HMGA2 as a chromatin architectural regulator, we performed 3C assays to analyze spatial interactions within the *MMP1*, *MMP2*, and *MMP9* promoter regions. Using R1 of *MMP1* as the PCR anchor, we detected strong chromatin conformation enrichment between R1 and TSS, which was attenuated by HMGA2 knockdown (**Figure 6B**, **Supplementary Figure 3A**). Reciprocal analysis using TSS as the anchor confirmed chromatin conformation enrichment to R1 (**Figure 6C**, **Supplementary Figure 3B**). Similar spatial organization patterns were observed for *MMP2*, with R2 as the anchor, revealing enrichments among R1, R2 and the TSS (**Figure 6D**, **Supplementary Figure 3C**), and R1 was the anchor that interacted with R2 and the TSS (**Figure 6E**, **Supplementary Figure 3D**) and TSS as the anchor, demonstrating enrichments with both R1 and R2 (**Figure 6F**, **Supplementary Figure 3E**). Consistent chromatin conformation enrichments patterns were also detected in the *MMP9* promoter region (**Figures 6G–I**, **Supplementary Figure 3F-H**).

**Figure 7:**
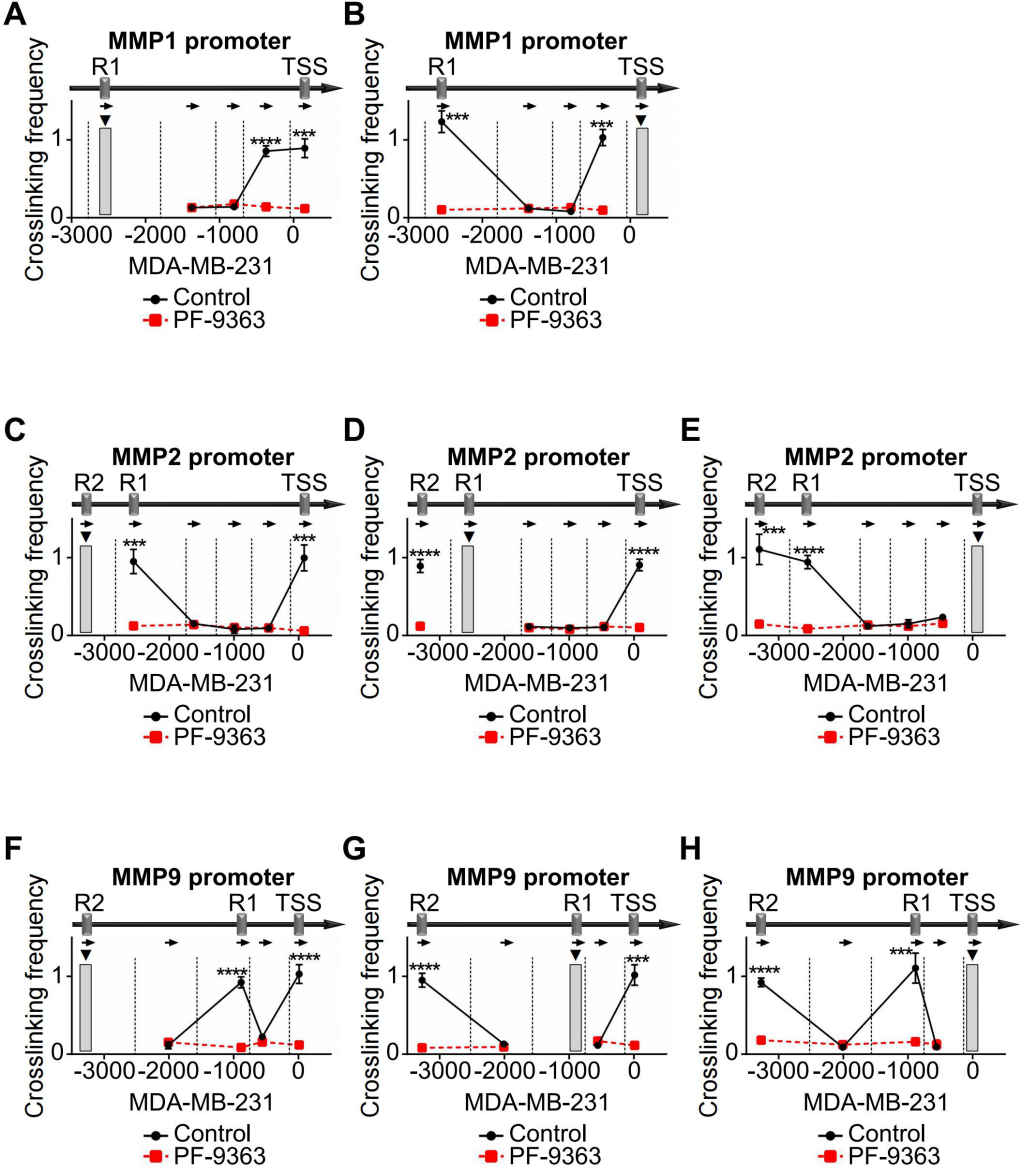
PF-9363 attenuated chromatin conformation enrichments in *MMPs* promoters. **(A, B)** In MDA-MB-231 cells, treatment with PF-9363 blocked the formation of R1/TSS cross-linking in *MMP1* promoter. **(C-E)** In MDA-MB-231 cells, PF-9363 treatment dissociated cross-links among TSS, R1 and R2 in *MMP2* promoter. **(F–H)** In MDA-MB-231 cells, PF-9363 departed cross-links among TSS, R1 and R2 in *MMP9* promoter. ***p<0.001, ****p<0.0001.

In addition, we used KAT6A inhibitor PF-9363 in TNBC cells to block KAT6A activity and performed 3C assays to detect the chromatin conformation changes in the promoter regions of *MMPs*. After treatment with PF-9363, the chromatin conformation enrichments between TSS and R1 in *MMP1* promoter were attenuated compared with control (**Figures 7A, B**, **Supplementary Figures 4A, B**). In *MMP2* promoter, the PF-9363 treatment blocked the chromatin conformation interaction forming among TSS, R1 and R2 (**Figures 7C–E**, **Supplementary Figures 4C–E**). In *MMP9* promoter, a similar effect could also be observed (**Figures 7F–H**, **Supplementary Figures 4F–H**). In PF-9363 treatment group, the chromatin conformation enrichments of different *MMPs* promoter regions were similar as the effects caused by HMGA2 knocking down. Collectively, these findings demonstrated that HMGA2 recruits KAT6A to *MMPs* promoters and induces histone acetylation and chromatin conformation changes, ultimately activating MMPs transcription.

### 3.7 HMGA2 promotes TNBC cells migration and invasion via MMPs

**Figure 8:**
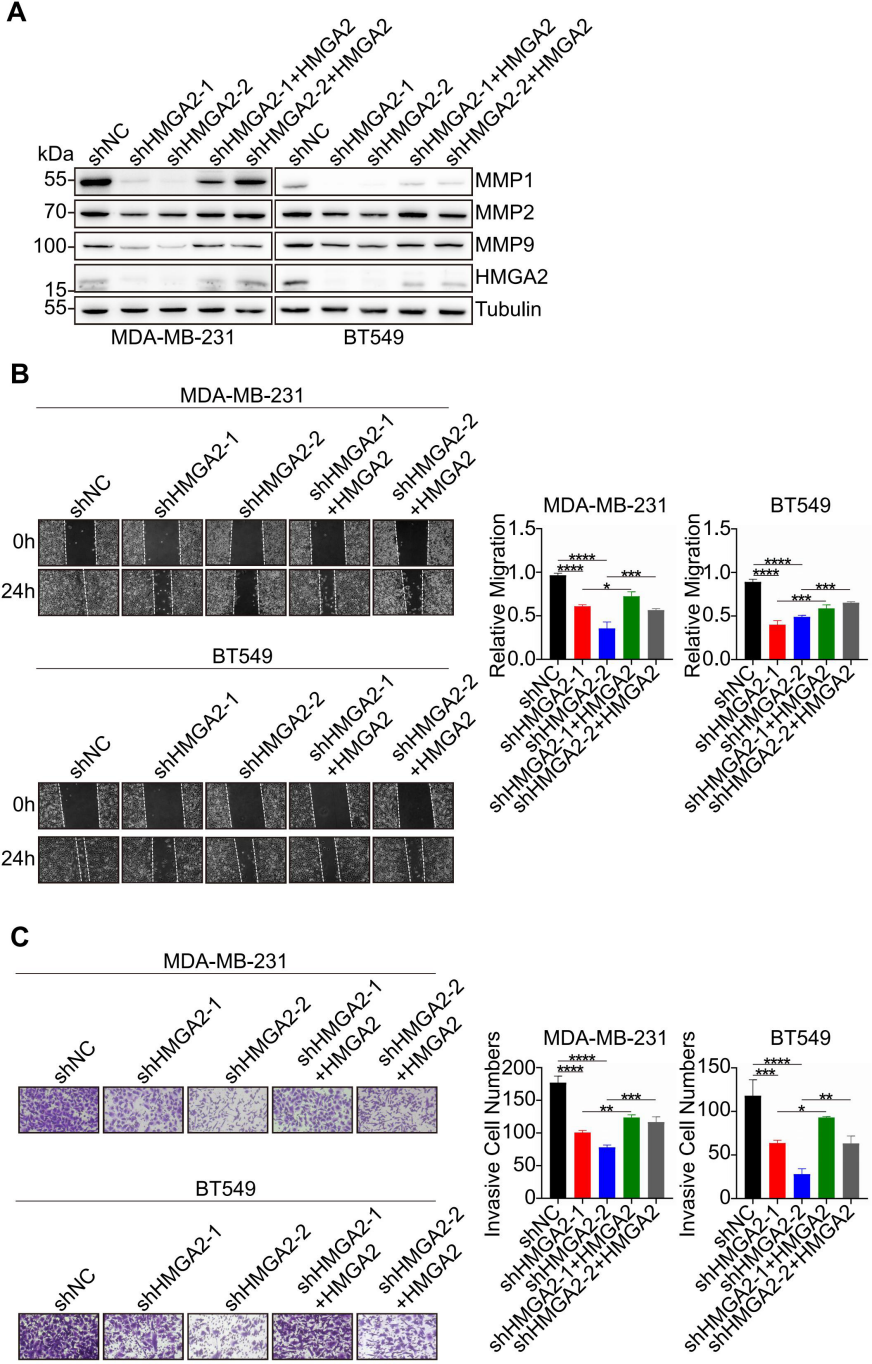
HMGA2 promotes TNBC cells migration and invasion via MMPs. **(A)** WB data of MMPs expression levels in TNBC cells of shNC group, two HMGA2 knocked down groups (shHMGA2-1, shHMGA2-2), and two HMGA2 rescued expression groups (shHMGA2 + HMGA2 res). **(B, C)** The wound healing and transwell assays detected aforementioned TNBC cells migration and invasion. *p<0.05, **p<0.01, ***p<0.001, ****p<0.0001.

To validate HMGA2’s functional role in TNBC cells, we restored its expression in shHMGA2 groups, which resulted in a corresponding increase in MMPs protein levels (**Figure 8A**). Furthermore, HMGA2 re-expression enhanced TNBC cells migration and invasion (**Figures 8B, C**).

## 4 Discussion

TNBC is an aggressive subtype of breast cancer with limited treatment options and a lack of effective therapeutic targets (27). Although previous studies have implicated HMGA2 in TNBC metastasis (12), the precise mechanisms by which it regulates chromatin conformation changes remain unclear. In this study, we identified HMGA2 as a critical architectural regulator of TNBC metastasis. Our findings revealed that HMGA2 expression is significantly elevated in TNBC tissues than in non-TNBC tissues. Furthermore, up-regulated HMGA2 expression correlated with poor prognosis in patients with TNBC, thus highlighting its potential as a prognostic biomarker. Functional studies using loss-of-function approaches confirmed HMGA2’s essential role in TNBC metastasis. Mechanistically, we revealed that HMGA2 interacts with KAT6A to regulate MMPs transcription, thereby promoting TNBC metastasis. Additionally, our findings showed that HMGA2 modulates chromatin conformation changes at *MMPs* loci. Collectively, these results suggested that HMGA2 may act as a novel biomarker for TNBC metastasis.

Alterations in histone modifications are fundamental epigenetic events that accompany transcriptional regulation and serve as critical drivers of oncogenesis and cancer progression. The N-terminus of KAT6A contains a double plant homeodomain (PHD) zinc finger domain and MYST domain, and the C-terminus of KAT6A contains a glutamate/aspartate-rich region and a serine/methionine-rich region (28). Considering that the MYST domain possesses acetyltransferase activity, and the serine/methionine-rich region possesses transcriptional activation activity (15), KAT6A can regulate chromatin structure and function by catalyzing the acetylation of specific amino acid residues, thereby affecting gene expression (14). KAT6A was previously established as an oncogene in TNBC through its interaction with SMAD3, which mediates TRIM24-SMAD3 complex recruitment to chromatin. Additionally, in TNBC immune microenvironment, the KAT6A/SMAD3/IL6/CD163 molecular axis drives macrophage M2 polarization (29). However, the role of KAT6A in TNBC requires further investigations.

Chromatin structure is crucial for gene expression and regulation (30, 31). For instance, the homolog family member A (*RhoA*) promoter contains two staphylococcal nuclease domain-containing protein 1 (SND1) recognition sites, where SND1 facilitates long-range chromatin interactions while linking histone H3K9ac to chromatin remodeling (32). SND1 promotes DNA methyltransferase 3 alpha (DNMT3A) transcription by modulating long-range chromatin remodeling of the *DNMT3A* promoter (26). Additionally, the transcription factor MYC regulates prostate cancer-specific gene transcription by remodeling CTCF-mediated chromatin structure (33). These results highlight the significance of chromatin conformation changes in tumorigenesis.

**Figure 9:**
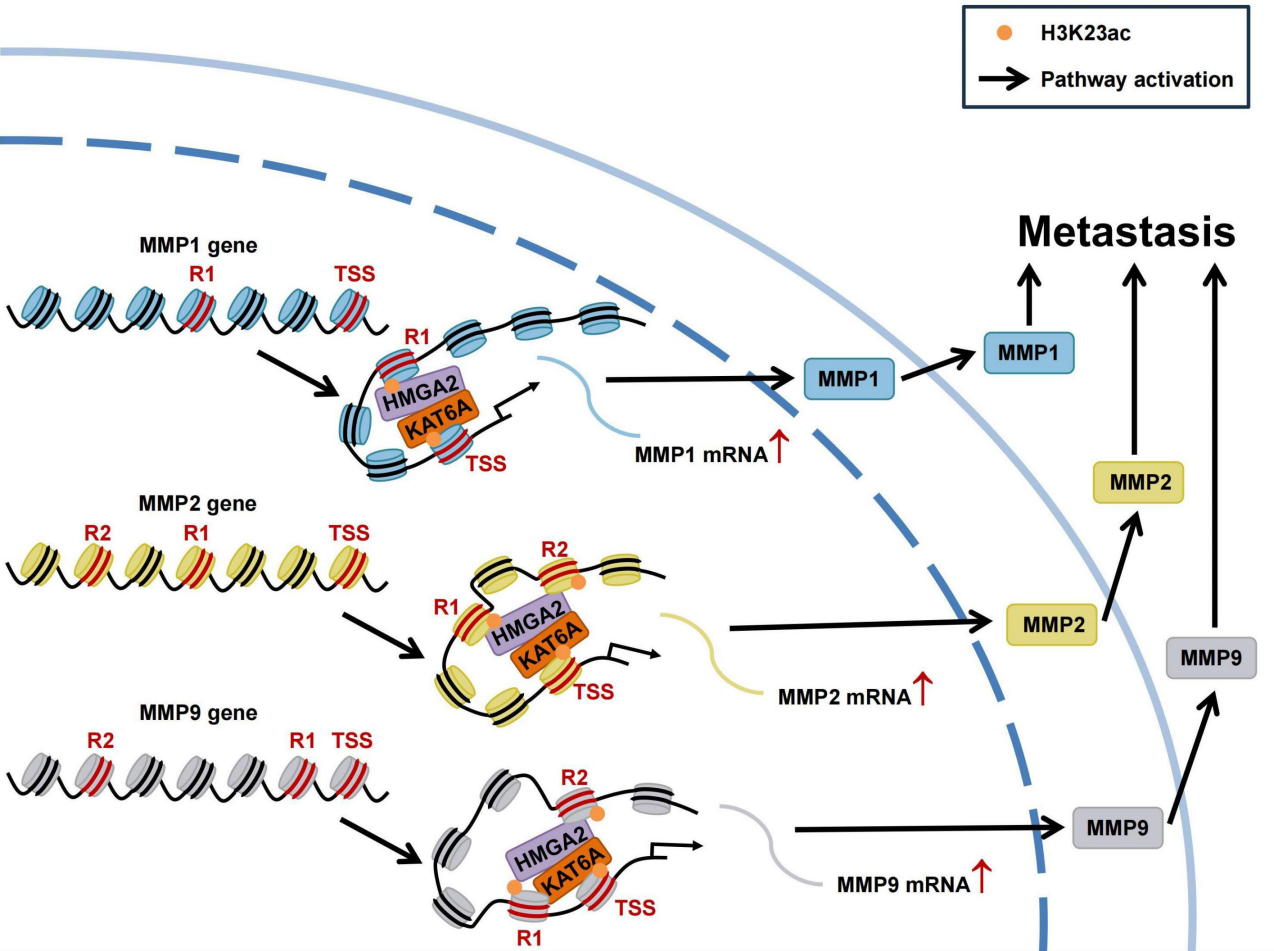
Schematic of HMGA2 interacts with KAT6A to regulate MMPs chromatin architecture and promote triple-negative breast cancer metastasis. HMGA2 directly binds to *MMPs* promoters to regulate their transcription. In addition, HMGA2 interacts with KAT6A to induce histone H3K23ac on *MMPs* promoters, accompanying with chromatin conformation changes. Extracellular matrix degradation induced by MMPs promote the metastasis of TNBC.

Overall, our study highlights HMGA2’s role as a chromatin architectural regulator in TNBC metastasis. Mechanistically, HMGA2 binds to *MMPs* promoters to regulate their transcription. Furthermore, HMGA2 interacts with KAT6A to catalyze H3K23ac modifications in *MMPs* promoter regions. These coordinated epigenetic modifications, along with chromatin conformation changes, ultimately promote MMPs transcription (**Figure 9**).

## Data Availability

RNA sequencing data in this study have been uploaded to the GEO repository (accession number GSE296619).

## Supplementary material

Supplementary Figure 1 The endogenous HMGA2 in TNBC cells. The endogenous HMGA2 were detected by IF in TNBC cells.

Supplementary Figure 2 HMGA2 regulates enzymatic activities of both MMP2 and MMP9. MMP2 and MMP9 enzymatic activities were detected by gelatin zymograohy assay.

Supplementary Figure 3 HMGA2 promotes MMPs transcription via chromatin conformation changes. **(A)** In BT549 cells, the anchor was R1 of *MMP1*, 3C data showed the DNA cross-link among R1 to TSS. HMGA2 knockdown attenuated the formation of R1/TSS cross-linking. **(B)** In BT549 cells, the anchor was TSS of *MMP1*, and the cross-link among TSS to R1 were shown. **(C, F)** In BT549 cells, the anchor was R2 of *MMP2* or *MMP9*, and the cross-links among R2 to R1 and TSS were shown. **(D, G)** In BT549 cells, the anchor was R1 of *MMP2* or *MMP9*, and the cross-links among R1 to R2 and TSS were shown. **(E, H)** In BT549 cells, the anchor was TSS of *MMP2* or *MMP9*, and the cross-links among TSS to R1 and R2 were shown. ****p<0.0001.

Supplementary Figure 4 PF-9363 attenuated chromatin conformation enrichments in *MMPs* promoters. In BT549 cells, PF-9363 attenuated the formation of chromatin conformation enrichments in *MMP1* **(A, B)**, *MMP2* **(C-E)** and *MMP9* **(F–H)** promoters. **p<0.01, ***p<0.001, ****p<0.0001.
