# Supplementary figures and images for: HMGA2 interacts with KAT6A to regulate MMPs chromatin architecture and promote triple-negative breast cancer metastasis
Source: Front Immunol. 2025 May 22;16:1590368. doi: 10.3389/fimmu.2025.1590368 (PMC12137317; doi:10.3389/fimmu.2025.1590368)

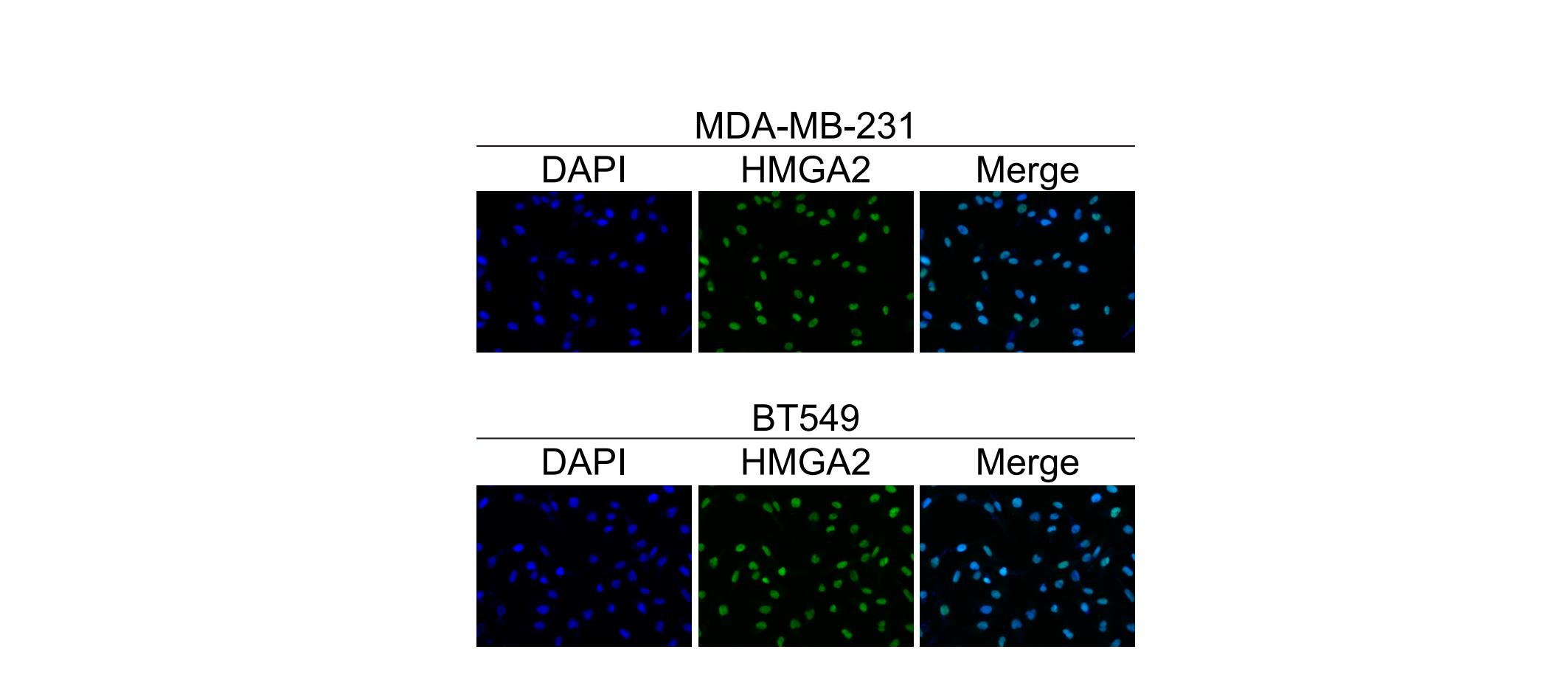

Supplement: Supplementary Figure 1 — The endogenous HMGA2 in TNBC cells. The endogenous HMGA2 were detected by IF in TNBC cells. [file Image1.jpg]

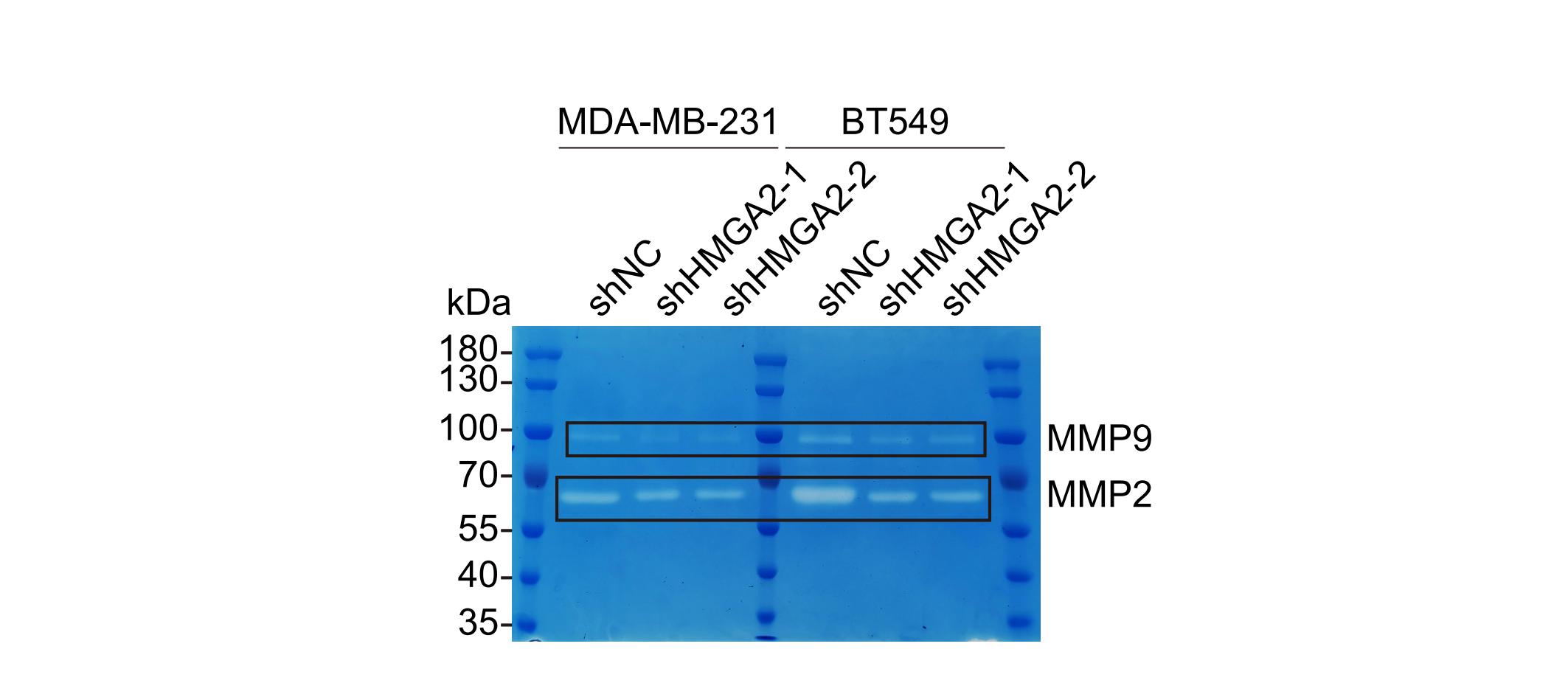

Supplement: Supplementary Figure 2 — HMGA2 regulates enzymatic activities of both MMP2 and MMP9. MMP2 and MMP9 enzymatic activities were detected by gelatin zymograohy assay. [file Image2.jpg]

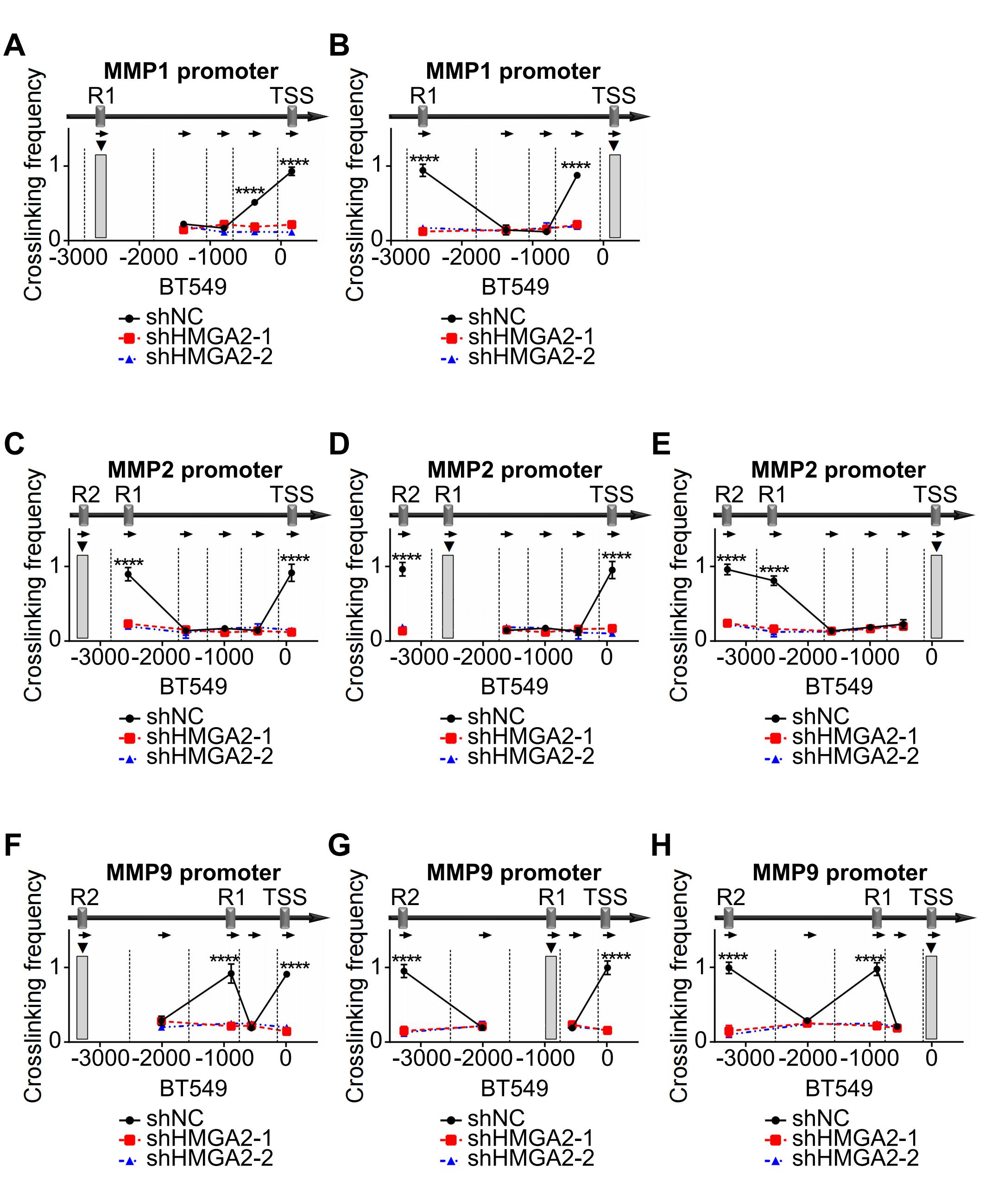

Supplement: Supplementary Figure 3 — HMGA2 promotes MMPs transcription via chromatin conformation changes. (A) In BT549 cells, the anchor was R1 of MMP1, 3C data showed the DNA cross-link among R1 to TSS. HMGA2 knockdown attenuated the formation of R1/TSS cross-linking. (B) In BT549 cells, the anchor was TSS of MMP1, and the cross-link among TSS to R1 were shown. (C, F) In BT549 cells, the anchor was R2 of MMP2 or MMP9, and the cross-links among R2 to R1 and TSS were shown. (D, G) In BT549 cells, the anchor was R1 of MMP2 or MMP9, and the cross-links among R1 to R2 and TSS were shown. (E, H) In BT549 cells, the anchor was TSS of MMP2 or MMP9, and the cross-links among TSS to R1 and R2 were shown. ****p<0.0001. [file Image3.jpg]

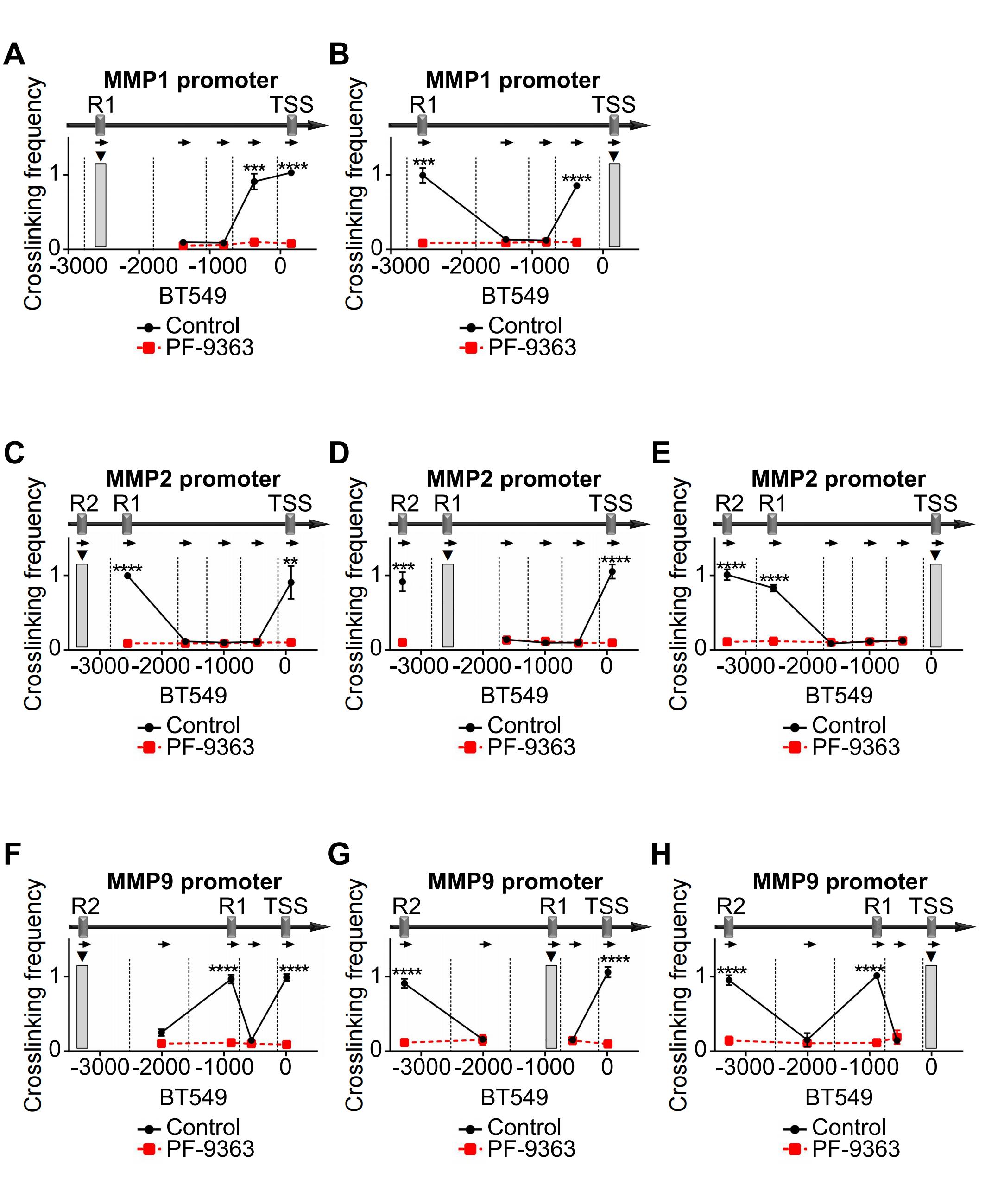

Supplement: Supplementary Figure 4 — PF-9363 attenuated chromatin conformation enrichments in MMPs promoters. In BT549 cells, PF-9363 attenuated the formation of chromatin conformation enrichments in MMP1 (A, B), MMP2 (C-E) and MMP9 (F–H) promoters. **p<0.01, ***p<0.001, ****p<0.0001. [file Image4.jpg]
