# Supplementary material for: HMGA2 interacts with KAT6A to regulate MMPs chromatin architecture and promote triple-negative breast cancer metastasis
Source: Front Immunol. 2025 May 22;16:1590368. doi: 10.3389/fimmu.2025.1590368 (PMC12137317; doi:10.3389/fimmu.2025.1590368)
Supplement: Supplementary file 5 [file Table1.docx]

Supplementary Material

# Supplementary Tables

**Supplementary Table 1.** The clinical features of patients in this study

| Feature | Normal (n = 6) | TNBC (n = 60) | Non-TNBC (n = 20) |
| --- | --- | --- | --- |
| Age (Year, Mean ± SD) | 55.2 ± 19.4 | 51.1 ± 5.7 | 56.4 ± 12.6 |
| TNM stage | | | |
| Stage Ⅰ | 0 | 3 | 3 |
| Stage Ⅱ | 0 | 12 | 8 |
| Stage Ⅲ | 0 | 27 | 9 |
| Stage Ⅳ | 0 | 18 | 0 |

Abbreviation: SD, standard deviation.

**Supplementary Table 2.** RT**-**qPCR primers used in this study

| RT**-**qPCR primers | | Sequence |
| --- | --- | --- |
| GAPDH | Forward | GTCTCCTCTGACTTCAACAGCG |
|  | Reverse | ACCACCCTGTTGCTGTAGCCAA |
| HMGA2 | Forward | GCGCCTCAGAAGAGAGGAC |
|  | Reverse | TTGAGCTGCTTTAGAGGGACTC |
| MMP1 | Forward | ATGAAGCAGCCCAGATGTGGAG |
|  | Reverse | TGGTCCACATCTGCTCTTGGCA |
| MMP2 | Forward | AGCGAGTGGATGCCGCCTTTAA |
|  | Reverse | CATTCCAGGCATCTGCGATGAG |
| MMP9 | Forward | GCCACTACTGTGCCTTTGAGTC |
|  | Reverse | CCCTCAGAGAATCGCCAGTACT |

**Supplementary Table 3.** ChIP primers used in this study

| ChIP primers | | Sequence |
| --- | --- | --- |
| MMP1-ChIP-(-3470 ~ -3270) | Forward | AGTGACAGCTTGAAGGGGAGGA |
|  | Reverse | GTTGAACACAGTTCTGTGCCAC |
| MMP1-ChIP-(-2432 ~ -2232) | Forward | AGGGCCATGTGAATCTAGGCTG |
|  | Reverse | TTCTCTAGCAAAGCTCCCTGCA |
| MMP1-ChIP-(-1133 ~ -933) | Forward | ATGCTGCCTAGCACCAAGGAGC |
|  | Reverse | CAGGCGGAGTATGAGATAACTC |
| MMP1-ChIP-(-101 ~ +99) | Forward | CATAGCTAATCAAGAGGATGTT |
|  | Reverse | GCAGCAGCAGCAGTGGAGGAAA |
| MMP2-ChIP-(-4845 ~ -4645) | Forward | ACTTTGTCAGTATCAACAATAA |
|  | Reverse | TACGTCAAAAGCTAGAAATGTC |
| MMP2-ChIP-(-3827 ~ -3627) | Forward | CACAAACCTTCAATTTGCAAAA |
|  | Reverse | CAATGTGACCCAAGCTGGGCTA |
| MMP2-ChIP-(-2665 ~ -2455) | Forward | ATGTGCTCACCAACAAGCCCAT |
|  | Reverse | CTTTTGGGGCTGATCTGCCGAT |
| MMP2-ChIP-(-1480 ~ -1280) | Forward | ACGTGGACATGAGCCCAGTGGG |
|  | Reverse | TGTCTGGTGGGTGTGTGCTCTG |
| MMP2-ChIP-(-108 ~ -92) | Forward | ATCTCTCTGTCCCTATCCCTAA |
|  | Reverse | AAACATCTGTCGCTCTAGGCCC |
| MMP9-ChIP-(-4728 ~ -4528) | Forward | GCTACCTCACTCTTATTTTCCT |
|  | Reverse | GCTAAGATCAGGTCACTGGCTC |
| MMP9-ChIP-(-3497 ~ -3297) | Forward | AGGTAAAGGAGGAAGTTGATGG |
|  | Reverse | TAAAGTGCCTGGCACATGATAA |
| MMP9-ChIP-(-2231 ~ -2031) | Forward | CTATACCTGGGTCATCACAGTT |
|  | Reverse | CTCACCACCTGTAAGGTAAGTT |
| MMP9-ChIP-(-1005 ~ -805) | Forward | GTATCCTTGACCTTCTTTCTGG |
|  | Reverse | GATTAAATGAGATCAAACATAT |
| MMP9-ChIP-(-92 ~ +108) | Forward | ACACCCTGACCCCTGAGTCAGC |
|  | Reverse | AGCACAAGGGTGGACTGGCGCT |

**Supplementary Table 4.** EMSA probes used for this study

| EMSA probes | Sequence |
| --- | --- |
| EMSA-Forward | 5’-ACGTTGCCATGGACGTTGCCATGGACATTGCCATGGACATTGCCATGG-3’ |
| EMSA-Reverse | 5’-CCATGGTAATGTCCATGGTAATGTCCATGGTAACGTCCATGGTAACGT-3’ |

**Supplementary Table 5.** 3C Primers used in this study

| 3C Primers | Sequence |
| --- | --- |
| MMP1-1 | TTCCATGAATACCTAACTGGA |
| MMP1-2 | TGAATGGAGGAATGCATAAAT |
| MMP1-3 | CTATTTGTCCCATGATAATGATG |
| MMP1-4 | GTGACTAGGACTACAGGTGC |
| MMP1-5 | TAGTCCAGGTAAATGCTGCATTGC |
| MMP2-1 | CTGAGACTAGAAGTCCAAAACCAAC |
| MMP2-2 | CAGGGCTCCCAGGGTTACCGTGGC |
| MMP2-3 | GAAAGCAAGTTTATTAAGAAAG |
| MMP2-4 | GGTACAGTCAAAGCTGCCCTCTGCCAG |
| MMP2-5 | GAGGCCACATGGATTCTTGGC |
| MMP2-6 | CAGCAGGGGGTTCTGAGGCTGTGC |
| MMP9-1 | GGCTATCTTGGTCACTACTTG |
| MMP9-2 | CCTTCAGTGGGTAGCAGGGGAC |
| MMP9-3 | CCTGGCCCTGAATCTTGGGTC |
| MMP9-4 | GACAGAGCCTGGAGTGTGGG |
| MMP9-5 | GTGGGGAAGGGGATGTGGTAACCA |
